# Acceptability of a Serious Game About Proton Radiotherapy Designed for Children Aged 5 to 14 Years and Its Potential Impact on Perceived Anxiety: Feasibility and Randomized Controlled Pilot Trial

**DOI:** 10.2196/54082

**Published:** 2024-09-23

**Authors:** Catarina Cederved, Gustaf Ljungman, Jon Back, Charlotte Ångström-Brännström, Gunn Engvall

**Affiliations:** 1 Department of Women's and Children's Health, Uppsala University, Sjukhusvägen, 751 85, Uppsala, Sweden; 2 Department of Neurobiology, Care Sciences, and Society, Karolinska Institutet, Stockholm, Sweden; 3 Department of Informatics and Media, Uppsala University, Uppsala, Sweden; 4 Department of Nursing, Umeå University, Umeå, Sweden

**Keywords:** anxiety, feasibility, acceptability, pediatric oncology, psychological preparation, proton radiotherapy, serious game, games, cancer

## Abstract

**Background:**

Children who are going to undergo radiotherapy have displayed fear and anxiety. Therefore, a web-based serious game was developed as a psychological preparation to investigate if it could affect anxiety levels. In an earlier stage, children with experience of radiotherapy had been part of the developmental process.

**Objective:**

The study aimed to investigate the feasibility in terms of reach, usability, and acceptability of a serious game about proton radiotherapy and to pilot that it did not increase anxiety levels in children aged 5 to 14 years undergoing radiotherapy.

**Methods:**

The design was a randomized controlled pilot trial with predefined feasibility criteria. In total, 28 children were assessed for eligibility, and 23 met the inclusion criteria. They were consecutively randomized into 1 of 2 study arms. One child was excluded after randomization. If randomized into arm 1, the children received the intervention before treatment started. Children in arm 2 were treated as controls. Questionnaires with fixed answers were used to assess anxiety levels (an adapted version of the State-Trait Anxiety Inventory for Children) and experiences of gameplay (an adapted version of Player Experience of Need Satisfaction [PENS]). The children were asked to answer questionnaires at 5 different measurement occasions during their radiotherapy treatment.

**Results:**

In arm 1, age ranged from 5 to 13 (mean 8.4, SD 2.4) years. In arm 2, age ranged from 5 to 11 (mean 7.6, SD 2.3) years. The sample consisted of 15 girls and 7 boys. The feasibility criterion that the children should play the game for 20 minutes or more was not met. Mean playtime for children in arm 1 was 32.1 (SD 23.8) minutes, where 18 children had played for at least 15 minutes. The criterion that 70% (n=16) or more of the participants should return all of the questionnaires was not met; however, more than 73% (n=16) returned the PENS questionnaires. The State-Trait Anxiety Inventory for Children was returned by 73% (n=16) on day 0, 77% (n=17) on day 1, 82% (n=18) on day 3, 82% (n=18) on day 6, and 86% (n=19) on day 15.

**Conclusions:**

All feasibility criteria set for the study were not met, suggesting that adaptions need to be made if a future study is to be undertaken. Further, the analysis revealed that there was no indication that playing increased the children’s self-reported anxiety. The PENS questionnaire adapted for children showed promising results regarding player satisfaction when using the serious game. When studying children with severe conditions and young age, 5 measurement occasions seemed to be too many. Measuring both player satisfaction or experience and knowledge transfer would be preferable in future studies.

## Introduction

### Background

Children with cancer are subjected to medical procedures that are both tiresome and painful [1]. Some children need to undergo radiotherapy. The treatment does not hurt but can be perceived as frightening [2]. Radiotherapy is usually given over a longer period of up to 6 weeks [3]. When radiotherapy is performed, the children are left alone in the enormous treatment room [4]. Since the children need to be completely still during the procedure, they are also fixated to stay immobilized [4,5]. When radiotherapy is targeted at the head, a mask is made and firmly fixated to the table [6]. The experience has been described as stressful and invoking anxiety and fear in both the children undergoing radiotherapy and their parents [7,8]. Several of the children are sedated, especially preschool children [2] because of the anxiety the procedure induces in the children [9]. Informing and preparing children who are going to undergo radiotherapy in order to decrease sedation or anesthesia (hereafter mentioned as sedation) or anxiety levels have been studied, and the effects have been minor [10,11]. However, with extensive psychological preparation, a decrease in the number of sedations was reported by Clerici et al [12]. An increased proportion of proton beam therapy is given as the preferred irradiation type, as it lessens short- and long-term toxicities and improves the quality of life outcomes for the children [13]. To receive proton therapy, the family often needs to go to a clinic far away from their home since only a few clinics provide such treatment [14,15]. In Sweden, where the study was executed, there is one national clinic providing proton beam therapy. Leaving their familiar surroundings can lead to further stress [16] on top of the pain and distress already caused by other cancer-related procedures the child has to endure [17]. It can therefore be argued that it is necessary to prepare the children by informing them about what radiotherapy is, what they can expect from the treatment that they are going to receive, and introducing them to the environment of the clinic so that they can feel that they are as much in control of the situation as possible [18].

In school, educational games have been proven to affect children’s learning [19]. Games can be enjoyable, create engagement, and induce behavior changes [20]. Using digital tools and games to prepare children before procedures and having them take part in the development process are becoming more common [21-23]. Children with cancer have suggested that information should be given to elevate understanding about a specific treatment, which also encourages their ability to cope with the procedure [24]. Knowledge about the procedure increased, and the level of anxiety decreased with a digital tool when tested on children who were to undergo planned hospital procedures [25]. Several games have been developed with the intent to increase knowledge about the disease and treatment for children with cancer [26]. To decrease anxiety, a serious game about proton radiotherapy was developed together with children to function as interactive information before the procedure [23]. However, how well the game functions needs to be evaluated. Therefore, as psychological preparation before radiotherapy, a serious game as a supplement to preparation already in place at a proton clinic was evaluated for acceptability and impact.

### Aim

The aim of the study is to investigate the feasibility in terms of reach, usability, and acceptability of a serious game about radiotherapy and to pilot that it does not increase self-reported anxiety in children aged 5 to 14 years undergoing radiotherapy.

## Methods

### Study Design

The study was designed as a feasibility and randomized controlled pilot study. The study provided an intervention using a prospective, waiting list control design.

### Serious Game

**Figure 1.**
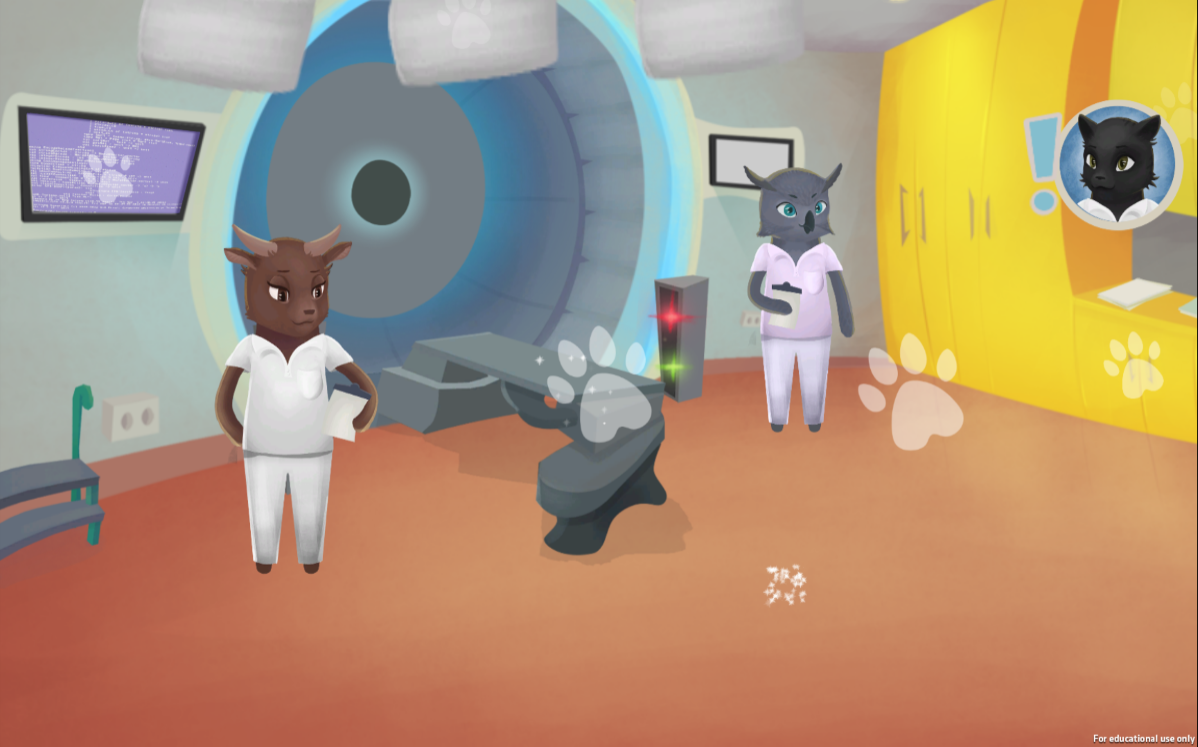
Screenshot from the game the participants were subjected to within the randomized controlled trial showing the first encounter of where radiotherapy is performed and where the avatar will later undergo radiotherapy.

For the intervention, children who would be undergoing radiotherapy played a web-based serious game on their tablet or computer to familiarize themselves with the radiotherapy treatment. The parents received a link to the game via email so that the children could access the game whenever they wanted. They used their own devices to play the game; however, they could also use the computers at the hotel they stayed at in conjunction with the treatment. No game controller was used, instead, the game was played with the computer’s mouse or by touching the tablet’s touchscreen. The game was designed as a doll house experience [27] where the children played an avatar that was in a proton radiotherapy clinic. In the game, a map was used that the child could click on to access different rooms in the clinic to explore them independently. The rooms contained information about the procedure of radiotherapy for educational purposes as displayed in Figures1  2. It also contained mini-games and game elements that were placed there to make exploration of the play setting interesting [23]. The game had been developed through an iterative process together with 9 children with experiences of radiotherapy treatment, which influenced the game’s design and led to numerous changes in it [23]. The changes pertained to the design of how the treatment was displayed and explained within the game and the language [23]. To be able to explore the clinic’s facilities and get informed about the procedure of radiotherapy is a form of psychological preparation [28]. The purpose of psychological preparation is to mitigate the fear of the unknown [29] and is intended to reduce anxiety before procedures [30].

**Figure 2.**
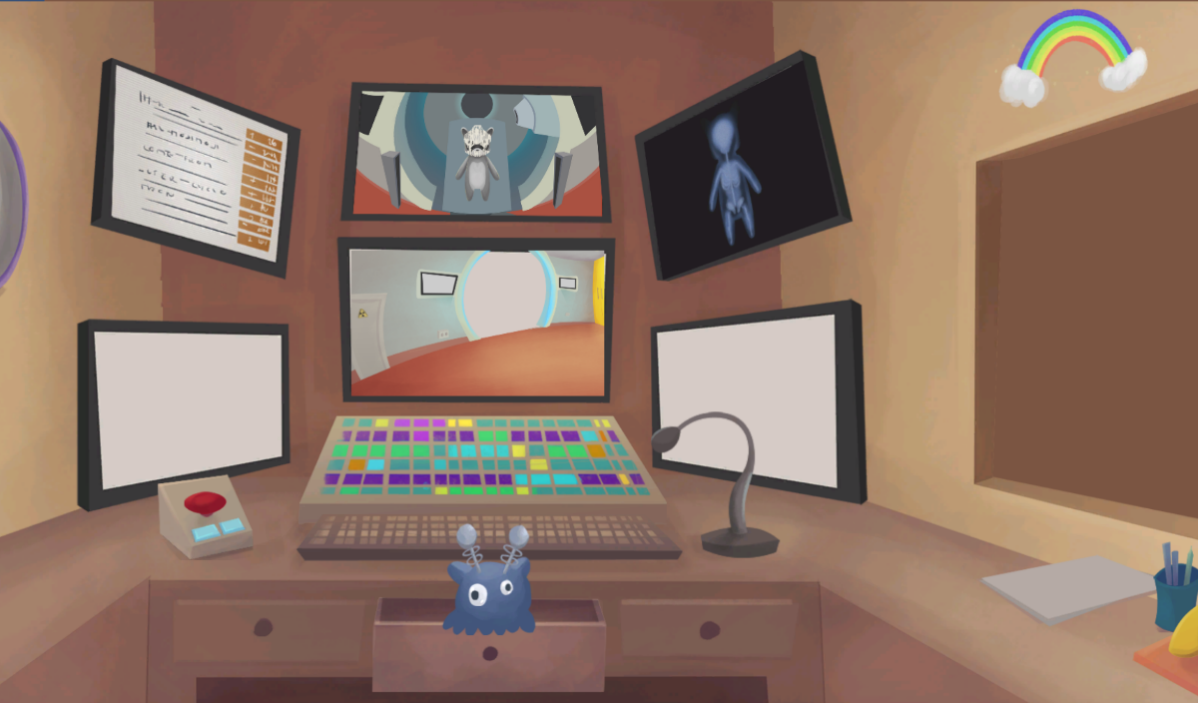
Screenshot from the game the participants were subjected to within the randomized controlled trial showing the monitor room where the staff provide therapy and observe the avatar undergoing radiotherapy.

### Recruitment

Recruitment started in February 2021 and completed in August 2022. During that period, 26 children were considered eligible for inclusion. When children between the ages of 5 and 15 years, living in Sweden, had been scheduled to have radiotherapy at the clinic, a written invitation about the study was sent to them by post. A few days later, a pediatric or oncology nurse or a coordinator contacted the family by telephone with information about the study and asked for permission for the researcher to contact them. Upon agreement, the researchers contacted them and gave further information orally, and if oral consent was granted, they were then emailed all further information about the study. Exclusion criteria were children living in countries other than Sweden, inability to understand Swedish, and severe mental disability. Two children were excluded due to language barriers during recruitment.

The children were divided into 3 age groups: ages 5‐7, 8‐10, and 11‐14 years. They were then randomly assigned into 1 of 2 study arms using stratified randomization in blocks to achieve balanced groups. The parents received information about which arm their child had been randomized to. Study arm 1 received the intervention 1 or more days before starting their therapy. Children assigned to arm 2 received the game 3 days after they started their therapy since it was not considered ethical to invite the children to a game study and then not let them play the game [31].

### Ethical Considerations

Ethics approval was received by the Swedish Ethical Review Authority (2020‐05578) before the study was started. The study was registered at ClinicalTrials.gov (NCT04728555). Upon agreement to participate in the study, the children received a 6-digit participation ID, which was used to sign into the game to be able to track time played, and the number was also used as identification for the questionnaires; hence, the data became anonymous. There was one separate list where participants’ names and contact information were kept together with their participation ID. The children’s legal guardian signed a written consent form on their child’s behalf and their own behalf. Children also gave their assent to participate. Neither the children nor their guardians received any compensation for participating in the study.

### Feasibility Criteria and Measurements

The feasibility criterion for success was that 80% (n=18) of the participants had played the game for 20 minutes or more. Further, 70% (n=17) or more of the participants should have returned all questionnaires partially or fully answered State-Trait Anxiety Inventory for Children-State Anxiety (STAIC(S)) and Player Experience of Need Satisfaction (PENS). The premises of the threshold were set to find out which days most children filled out the STAIC(S) to establish for future studies how many measurement occasions are plausible. A hypothesis was tested, which was that children in arm 1 should not communicate more anxiety than children allocated to arm 2. In addition, reach was measured by assessing whether prospective participants received information about the study.

There were 4 different questionnaires distributed to participating children: the STAIC(S) and State-Trait Anxiety Inventory for Children-Trait Anxiety [32], the PENS [33], and a customized questionnaire about radiotherapy developed by the research team (which will be reported elsewhere). Parents answered an adult version of the State-Trait Anxiety Inventory (STAI), which included 10 questions on a 4-point Likert scale [34]. They also answered a questionnaire providing background variables. Children who were not able to read were helped by their parents to answer the questionnaires and interpret the scale alternatives.

An adapted version of the short STAIC with a 3-point Likert scale was used to measure the children’s anxiety [32]. The short form of STAIC consists of 6 questions; however, 2 questions were added from the long version of STAIC to provide further insight into anxiety, making a range from 8 to 24 points. Hereafter, the adapted version used in the intervention will be referred to as STAIC(S). According to the guidelines of interpretation, a calculation based on 8 questions indicated 12 points as the cutoff for anxiety. To not overestimate the children’s anxiety, the cutoff was set at 13 points for feeling anxious. The State-Trait Anxiety Inventory for Children-Trait Anxiety is a questionnaire consisting of 10 questions on a 3-point Likert scale [32].

The PENS questionnaire consists of 16 questions on a 7-point Likert scale [33]. Approval was given for the questionnaire to be translated into Swedish and adapted for children by the owner company, Immersyve. It was first translated from English into Swedish by a group consisting of 2 pediatric nurses, 1 researcher in informatics and media, and 1 pediatric psychologist and then backward by an interpreter with English as their mother tongue. Two questions were related to multiplayer games, which were not relevant to the study since the game is a single-player game, so they were excluded. The questionnaire was tested for face validity on 4 healthy children between the ages of 5 and 11 years through interviews. The questionnaire was made in 2 versions, 1 for younger children (5‐7 years) and 1 for older children (8‐14 years). The younger population had difficulty understanding the 7-point Likert scale. Therefore, it was changed to a 3-point Likert scale. Furthermore, the language was adapted to the young population and consisted of 8 questions (range 8‐24). For the older children (8‐14 years), the questionnaire consisted of 14 questions (range 14‐98). The cutoff level for satisfaction with the game experiences was set at 16 for PENS (5‐7 years) and at 65 for PENS (8‐14 years), reaching two-thirds of the total score (66%) for both scales. The fourth questionnaire (customized questionnaire about radiotherapy) included 6 questions about radiotherapy on a 4-point Likert scale.

### Data Collection

At 1‐3 days prior to the start of treatment, the children randomized into arm 1 (intervention) received a web link to the game, and children randomized into arm 2 (control) received the web link after their third day of treatment. The children and parents received instructions stating that they were free to play the game as much or little as they liked. Each participant was given a unique participation number sent to their guardian’s email, which was used to access the game. A software engine was used to store the playtime for each number, and the data were deleted after a month. A member of the team who only had the participation number collected the data from the engine. The playtime was measured cumulatively, and no record was kept on how many times each participant had chosen to access the game. For 2 players, the information was lost. Therefore, the parents were emailed to make an estimation of their children’s playtime. Children in both arms answered the same questionnaires 1 day before treatment started (day 0), the first day of treatment, the third day of treatment, the sixth day of treatment, and the last questionnaire approximately 15 (±4) days into treatment. The children received a diary notes form on their first day at the clinic where they were asked open questions about how they perceived the game, which will be reported elsewhere. The questionnaires were administered to the children and parents by the receptionist staff at the clinic before they were due to have the treatment. The answered questionnaires were left in a mailbox at the clinic. The children and parents were asked to arrive before their appointment on the days of the study so that they would have time to answer the questionnaires before that day’s treatment. The total time children spent playing the game was collected digitally for each participant. STAIC(S) was collected on all 5 measuring occasions, PENS at 1 time, and radiotherapy questionnaire on 4 occasions. Parents answered demographic questions and STAI at the first measuring point. During participation in the study, the children and their parents received information according to standard care.

### Data Analysis

Descriptive statistics were used to gain insights into the collected quantitative data. To analyze differences between the arms, Fisher exact test, Mann-Whitney *U* test, Pearson chi-square, and Wilcoxon matched-pair signed rank were used. Spearman rank order correlation was used to analyze statistically significant associations between variables within the sample. In addition, Cronbach α was used to calculate the internal consistency between concepts. SPSS (version 28.0; IBM Corp) was used for the analyses, and the findings were considered to be statistically significant if a *P* value <.05 was reached. The CONSORT (Consolidated Standards of Reporting Trials) guideline was followed for statistical analysis and to report the study [35].

## Results

### Feasibility in Terms of Reach and Background Variables

**Figure 3.**
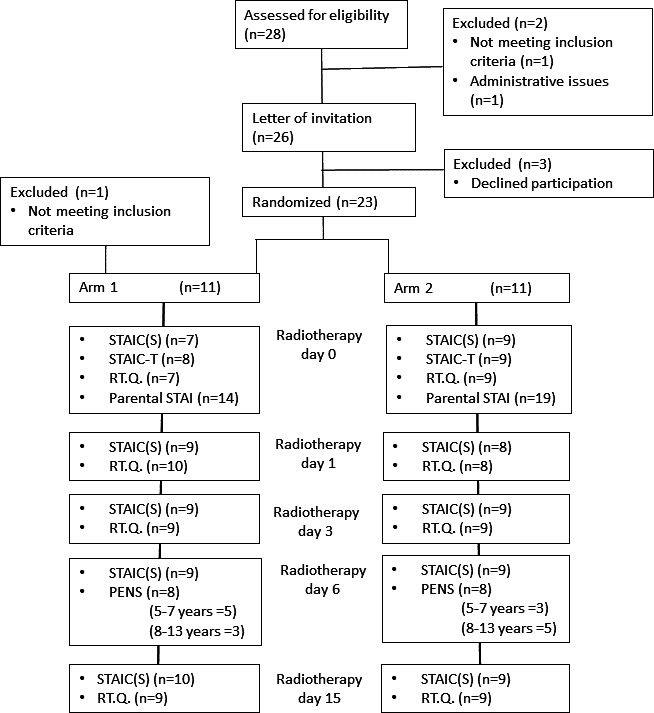
CONSORT (Consolidated Standards of Reporting Trials) flowchart depicting enrollment and the days the questionnaires were administered and how many participants returned them for each arm. PENS: Player Experience of Need Satisfaction; RT.Q.: customized questionnaire about radiotherapy; STAI: State-Trait Anxiety Inventory; STAIC(S): State-Trait Anxiety Inventory for Children-State Anxiety; STAIC-T: State-Trait Anxiety Inventory for Children-Trait Anxiety.

The study aimed to reach all patients within the age group who were to have radiotherapy. After the study had been finished, the nurses at the radiotherapy clinic went through the records of all children who had passed the clinic within the study’s timeframe. There had been 28 children who had been assessed for inclusion, of which, 26 of the children were eligible to take part in the study (Figure 3). A total of 3 children declined participation, and 1 child was lost to participation due to administrative issues, resulting in 22 (85%) children’s completion of at least one questionnaire of the study. There were no differences between the intervention arm and controls regarding sex, age, diagnosis, sedation, and parents’ educational level.

In total, 11 children were randomized into the intervention arm, and another 11 into the control arm. In arm 1, age ranged from 5 to 13 (mean 8.4, SD 2.4) years. In arm 2, age ranged from 5 to 11 (mean 7.6, SD 2.3) years. For the variances of ages, see Table 1. The sample consisted of 15 girls. A majority of the children (n=16) were diagnosed with brain tumors and 6 with extracranial solid tumors. The number of fractions received varied from 14 to 33 with a mean of 27 (SD 5.7) fractions. In total, 11 children received sedation, and 6 of these children received the intervention. All 5- and 6-year-old children were sedated, and 3 of 4 children among the 7-year-olds were also sedated. The sedated children’s answers are included in the analysis. Most of the children (n=21) lived with 2 parents, while 1 child lived with a single parent. A total of 16 children lived with a parent who had attended higher education and 4 lived with a parent who had finished high school, and there were 2 children for whom no data were attained.

**Table 1.** Children’s (n=22) background variables divided by allocated arm. Displaying number of children’s sex, age groups, sedated or awake status during radiotherapy, diagnosis, and parental education level with the total amount of each arm for each variable and total when merging the 2 arms.

		Arm 1, n (%)	Arm 2, n (%)	Total, n
**Sex**				
	Boy	2 (18)	5 (45)	7
	Girl	9 (82)	6 (55)	15
	Total	11	11	22
**Age groups (years)**				
	5‐7	5 (50)	5 (50)	10
	8‐10	4 (44)	5 (56)	9
	11‐14	2 (67)	1 (33)	3
	Total	11	11	22
**Sedated**				
	Sedated	6 (55)	5 (45)	11
	Awake	5 (45)	6 (65)	11
	Total	11	11	22
**Diagnosis**				
	Brain tumor	8 (50)	8 (50)	16
	Extracranial solid tumor	3 (50)	3 (50)	6
	Total	11	11	22
**Level of parents’ education**				
	Higher education	9 (56)	7 (44)	16
	High school	1 (25)	3 (75)	4
	Total	10	10	20

### Feasibility in Terms of Usability and Acceptability of the Intervention

The predefined feasibility criterion was that 80% (n=18) of the children should play for at least 20 minutes to determine whether they found the game acceptable. There were 6 (27%) participants who played the game for less than 20 minutes, and 18 (82%) of the participants played for more than 15 minutes; hence, the criterion was not fulfilled. However, the mean time playing was 31 minutes for the entire sample (median of 27 minutes) spanning from not playing the game at all to playing for 85 minutes (Table 2). The intervention arm had a mean time playing the game of 32.1 (SD 23.8) minutes, while the mean time was 29.9 (SD 19.0) minutes for the control arm. There were no differences in playtime between the two.

To measure the participants’ self-rated anxiety, the STAIC(S) was administered at 5 measurement points. The estimation was that 70% (n=17) of the participants should return the forms partially or fully completed. In total, 9 (41%) children returned all 5 forms of STAIC(S), and 13 (59%) children failed to return 1 or more forms. To measure the usability of the game, their experiences of the game were measured through the 2 versions of the PENS questionnaire, which 16 (73%) participants returned. A total of 8 participants answered the 5‐7 years version, and 8 participants answered the PENS questionnaire (8‐14 years).

**Table 2.** Description of children’s playtime of the serious game, Player Experience of Need Satisfaction (PENS) score, and if the child was sedated or awake during radiotherapy (n=22).

Participant	Age (years)	Time played (minutes)	PENS score	Sedated
**Intervention^a^**				
E1	5	29	11^b^	Sedated
E2	7	18	19^b^	Sedated
E3	7	20	18^b^	Awake
E4	7	58	19^b^	Sedated
E5	7	85	18^b^	Sedated
E6	8	10	—^c^	Sedated
E7	8	23	45^d^	Awake
E8	8	27	70^d^	Sedated
E9	10	45	—	Awake
E10	12	0	—	Awake
E11	13	38	67^d^	Awake
**Control** ^e^				
L1	5	0	—	Sedated
L2	5	10	—	Sedated
L3	5	20	16	Sedated
L4	5	60	22	Sedated
L5	6	23	20	Sedated
L6	9	17	34	Awake
L7	9	35	66	Awake
L8	9	38	—	Awake
L9	9	60	88	Awake
L10	10	39	47	Awake
L11	11	27	26	Awake

a Age: mean 8.4 (SD 2.4) years and time played: mean 32.1 (SD 23.8) minutes.

b PENS (children 5‐7 years) score range 8‐24, mean 17.9 (SD 3.3) based on both arms (n=8).

c Missing value.

d PENS (children 8‐14 years) score range 14‐98, mean 55.4 (SD 20.8) based on both arms (n=8).

e Age: mean 7.6 (SD 2.3) years and time played: mean 29.9 (SD 19.0) minutes.

### Anxiety

To measure whether children who received the intervention communicated an increase in anxiety compared to the controls on their first day of radiotherapy, the Mann-Whitney *U* test was used. No perceived differences between the arms could be found (*P*=.81). There were no differences measured in anxiety between the arms across all 5 assessment occasions.

The number of children who indicated anxiety (13‐24 points) through the STAIC(S) is presented in Table 3, where the children receiving the intervention and the control arm are displayed separately. There were 6 children sedated in the intervention arm and 5 in the control. The following response rates at each measurement occasion were collected: 73% (n=16) on day 0, 77% (n=17) on day 1, 82% (n=18) on days 3 and 6, and 86% (n=19) on day 15 (Table 3). Over time, the children’s anxiety decreased, but no significant difference could be found between the arms. There was no significant correlation found between STAIT (trait anxiety) and STAIC(S) (state anxiety) in the current sample (*P*=.34). The items’ internal consistency of STAIC(S) was calculated by Cronbach α based on 89 questionnaires that had been obtained during the study, and the α value was 0.85.

On day 0, the parental STAI questionnaire was administered to 43 parents, and 33 answers were obtained. The hypothesis was that if the parents presented higher levels of anxiety, so would their children. Since there were considerably more mothers who returned the parental STAI, the hypothesis was only tested between mothers and their children. No correlation could be found between the mother’s reported trait anxiety levels and the children’s before treatment started. No correlation could be found between the mother’s state anxiety levels and those of their children.

**Table 3.** The number of children reporting anxiety (13‐24 points) through the State-Trait Anxiety Inventory for Children-State Anxiety (STAIC(S)) questionnaire at 5 measurement occasions (n=22).

Measurement occasions	Intervention			Total, n	Control			Total, n
	Awake, n (%)	Sedated, n (%)	Total, n		Awake, n (%)	Sedated, n (%)	Total, n	
Day 0 (n=16)	2 (29)	4 (57)	6	7	3 (33)	2 (22)	5	9
Day 1 (n=17)	1 (11)	4 (44)	5	9	4 (50)	2 (25)	6	8
Day 3 (n=18)	1 (11)	3 (33)	4	9	2 (22)	1 (11)	3	9
Day 6 (n=18)	2 (22)	3 (33)	5	9	1 (11)	1 (11)	2	9
Day 15 (n=19)	2 (20)	2 (20)	4	10	1 (11)	1 (11)	2	9

### Serious Game About Radiotherapy

No correlation between time playing the game and anxiety levels measured on day 1 was found in the intervention arm nor was it found when combining the 2 arms on day 6. To check the translated and modified PENS questionnaire’s items for internal consistency and accuracy, we used the concepts that were established in PENS (version 1.6). PENS (5‐7 years) consisted of 8 questions: 2 items related to competence, 2 items to autonomy, and 4 items to presence, with a sample unit of 8. Six respondents were girls. The Cronbach α score for competence was 0.89, autonomy 0.57, and presence 0.28. Descriptive statistics of the concepts for PENS (5‐7 years) are reported in Table 4. For PENS (5‐7 years), the score could range between 8 and 24, and the mean score was 17.9 (SD 3.3; Table 2). No correlation between the time playing the game and the scoring of the questionnaire for the PENS (5‐7 years) was established. In total, 7 (88%) of the participants had a score that was 16 or higher, indicating that they found the game experience satisfying (Table 2).

PENS (8‐14 years) consisted of 14 questions, and there were 4 items related to competence, 3 to autonomy, and 7 to presence, with a sample unit of 8. Descriptive statistics of the concepts for PENS (8‐14 years) are reported in Table 5. The Cronbach α score for competence was 0.85, autonomy was 0.87, and presence was 0.91. For PENS (8‐14 years), the score could range between 14 and 98, and the mean score was 55.4 (SD 20.8; Table 2). A correlation between the time playing the game and the scoring of the questionnaire for the PENS (8‐14 years) could not be found with a *P* value of .05. However, it is noteworthy that it resulted in a *P* value of .06. In total, 4 (50%) of the participants had a score that was 65 or higher, indicating that they found the game experience satisfying.

**Table 4.** The three concepts by subscales present in Player Experience of Need Satisfaction (5-7 years; calculated from replies by 8 participants).

Concepts	Scores from participants aged 5‐7 years on a scale ranging from 1 to 3		
	Min	Max	Mean
Competence	2.38	2.63	2.50
Autonomy	1.63	2.50	2.06
Presence	1.88	2.50	2.13

**Table 5.** The three concepts by subscales in Player Experience of Need Satisfaction (8-14 years; calculated from replies by 8 participants).

Concepts	Scores from participants aged 8‐14 years on a scale ranging from 1 to 7		
	Min	Max	Mean
Competence	3.89	6.11	5.22
Autonomy	3.78	4.78	4.11
Presence	2.63	4	3.20

## Discussion

### Principal Findings

Web-based games are easily accessible and can work as psychological preparation for children with cancer [26]. Preparing children who are going to undergo radiotherapy with the help of a serious game could be a means to make the experience somewhat less frightening. All 3 predefined feasibility criteria of the study were not met. Less than 80% of the children played the game for 20 minutes or more. More than 70% returned the PENS questionnaire. Less than 70% returned all the questionnaires of STAIC(S). Although, on the last 3 occasions of assessment, more than 80% of the questionnaires were returned. The results did not show that the serious game increased the children’s anxiety, and over the trajectory of the study’s time, there was a decrease in anxiety levels in the 2 arms. The study achieved its desired reach by recruiting through nurses who contacted children who were to visit the clinic. Randomization was also done according to the pilot study protocol.

### Anxiety

Since the sample size was small, no difference could be found in anxiety levels between the ones receiving the intervention and the control arm, which was similar to findings in earlier studies [10,11]. The study relies on the participating children being able to identify their own feelings and express them accordingly to the scale in the STAIC(S). The scale, in its full version, is one of those most used in research and has been found to be both valid and reliable among children with cancer [36]. Children need to have had experiences of feelings to have learned their meaning if they are to be able to label them with words on a questionnaire. [37]. Nevertheless, it is preferable that children themselves report their symptoms [38]. The STAIC(S) is not validated for younger children (5‐7 years), which means another instrument would be preferable; for example, the STAIC(S) redesigned and including pictures aimed at younger children could be an option [39].

The sample included children who were sedated. Children who are sedated are not at risk of not being still during radiotherapy [5]; nevertheless, they still can feel fear and anxiety concerning the procedure [40]. Therefore, to include their answers about how they perceived the game and rated their anxiety is valuable to report since few earlier studies have measured children’s self-rated anxiety when undergoing radiotherapy.

Since 2 items were added to the short form of STAIC (including 8 instead of 6 questions), Cronbach α was used to assess the internal consistency by reliability tests, and the α value was 0.85, which is considered to prove high internal consistency between the items [41].

### Playing Time and PENS Scores

The feasibility criteria of the study were not met since fewer children than predicted played the game for 20 minutes or more. The children were free to play the game as much or little as they liked, resulting in a few children not playing the game at all. However, the time spent playing the game was almost the same in the arms, indicating an interest in the game among the children participating in the study. Other studies have used various specified game dosages and a set time playing the intervention, which in some cases resulted in a correlation to statistically significant findings [42]. To not have a predetermined play time but instead monitor how much time children spend playing the game is a way to assess whether children find serious games acceptable [43]. Cronbach α was used to assess the internal consistency through reliability tests of the 2 adapted PENS questionnaires developed for the study of the concepts [41]. For PENS (8‐14 years), the reliability was high. However, the number of participants that the calculation was established for was low. Therefore, the questionnaire should be validated by factor analysis, and reliability tested on a larger sample of children in a Swedish context [44]. Further, the validation of the English version was conducted using university students [33]. For PENS (5‐7 years), the reliability was low for 2 of the concepts and high for 1. This could be an effect of the change from the 7-point Likert scale to a 3-point Likert scale and that it consists of fewer questions. Therefore, the PENS (5‐7 years) also needs to be validated for children in a Swedish context.

### Methodological Considerations

Following the intention-to-treat analysis, the 2 children who never opened or played the game are part of the analyzed material as they answered questions. For future studies, predetermined play time of the intervention could be a means to establish whether it has an effect on what the study measures [42] and exclude those not achieving predetermined play time in the analysis. Fewer children than predicted returned all of the STAIC(S) questionnaires. For future studies, fewer assessment point days including fewer questions would be preferred when researching the population. In addition, another option to evaluate the effect of the intervention could be to measure the number of sedations before and after the intervention. For the study to reach power, a power calculation was made showing that 60 children needed to be enrolled. A strength of the study was that the clinic involved has a nationwide uptake and therefore reached all children within the age groups. However, after 1.5 years, only 28 children had been assessed for eligibility. The study was run during the COVID-19 pandemic, which might have affected referrals to the clinic. Despite prospective inclusion, there were more girls than boys included, which could be due to chance on account of the small sample size. Future studies need to have an even sex distribution to ensure generalizability. A weakness in the study was that it was unable to reach the estimated goal for power; and therefore, the criterion of a difference in the anxiety levels between the arms could not be found. With a larger study sample size, the criterion might have been met. Therefore, collaboration with other proton clinics is warranted to obtain a larger sample. Hence, the evaluation of the feasibility of the study provided information on how to improve the study protocol for future studies.

Furthermore, it is not possible to know if the children chose to answer the questionnaires before the treatment or after. On the questionnaires, the child or parent wrote the date of treatment they were in; however, the date sometimes differed from the date in their treatment plan. Staff had on occasion missed handing out the questionnaires on the correct date. In the future, a more precise collection is warranted, for example, having the questionnaires on the web would be favorable [45], and reminders could increase response rates [46].

### Implications for Future Studies

Some lessons learned through the feasibility study are the following: make sure that children in the study population under investigation have the possibility to take part as planned. Time needs to be set off for answering questionnaires within the hospital procedure schedule. When studying children with severe health conditions, it is important to be mindful of how many questions is possible for them to answer before they run out of energy. To test if games do what they are intended for within medicine and indicate if they would be used, it would be preferable to measure both player satisfaction and knowledge transfer.

### Conclusions

All feasibility criteria set for the study were not met, suggesting that adaptions need to be made if a future study is going to be undertaken. It is preferable to do a feasibility study since it is a way to detect the advantages and disadvantages of the study protocol and to optimize future studies. There was no indication found that playing the serious game increased the children’s self-reported anxiety toward undergoing radiotherapy. The PENS questionnaire showed promising results regarding player satisfaction in the use of the serious game within health care. When serious games are used as interventions, it is necessary to evaluate player satisfaction in future studies, and for that reason, the PENS questionnaire for children needs to be validated. To avoid dropout from the study, it would be commendable to not have as many as 5 measurement occasions for a group of the current age.

## Supplementary material

10.2196/54082 Checklist 1 CONSORT-EHEALTH (version 1.6.1) checklist.

## Abbreviations

CONSORT: Consolidated Standards of Reporting Trials
PENS: Player Experience of Need Satisfaction
STAI: State-Trait Anxiety Inventory
STAIC: State-Trait Anxiety Inventory for Children
STAIC(S): State-Trait Anxiety Inventory for Children-State Anxiety
